# Liquid biopsy: one cell at a time

**DOI:** 10.1038/s41698-019-0095-0

**Published:** 2019-10-02

**Authors:** Su Bin Lim, Wen Di Lee, Jyothsna Vasudevan, Wan-Teck Lim, Chwee Teck Lim

**Affiliations:** 1 NUS Graduate School for Integrative Sciences & Engineering, National University of Singapore, Singapore, Singapore; 2 Department of Biomedical Engineering, National University of Singapore, Singapore, Singapore; 3 Singapore University of Technology & Design, Singapore, Singapore; 4 Division of Medical Oncology, National Cancer Centre Singapore, Singapore, Singapore; 5 Office of Academic and Clinical Development, Duke-NUS Medical School, Singapore, Singapore; 6 IMCB NCC MPI Singapore Oncogenome Laboratory, Institute of Molecular and Cell Biology (IMCB), Agency for Science, Technology and Research (A*STAR), Singapore, Singapore; 7 Mechanobiology Institute, National University of Singapore, Singapore, Singapore; 8 Institute for Health Innovation and Technology (iHealthtech), National University of Singapore, Singapore, Singapore

**Keywords:** Nanobiotechnology, Tumour biomarkers

## Abstract

As an alternative target to surgically resected tissue specimens, liquid biopsy has gained much attention over the past decade. Of the various circulating biomarkers, circulating tumor cells (CTCs) have particularly opened new windows into the metastatic cascade, with their functional, biochemical, and biophysical properties. Given the extreme rarity of intact CTCs and the associated technical challenges, however, analyses have been limited to bulk-cell strategies, missing out on clinically significant sources of information from cellular heterogeneity. With recent technological developments, it is now possible to probe genetic material of CTCs at the single-cell resolution to study spatial and temporal dynamics in circulation. Here, we discuss recent transcriptomic profiling efforts that enabled single-cell characterization of patient-derived CTCs spanning diverse cancer types. We further highlight how expression data of these *putative* biomarkers have advanced our understanding of metastatic spectrum and provided a basis for the development of CTC-based liquid biopsies to track, monitor, and predict the efficacy of therapy and any emergent resistance.

## Introduction

Despite the first report on breast circulating tumor cells (CTCs) in 1869,^1^ techniques for isolating these circulating biomarkers were only first described in 1960,^2,3^ and were gradually improved over the next 40 years. While much progress has been made with the albumin gradient method and FDA-approved CellSearch® system during the *first* generation of CTC research,^4^ the existence of heterogeneous CTC subpopulation highlighted the need to develop marker-independent isolation technologies.^5,6^ Since then, label-free techniques utilizing the principles of biophysical properties have been developing rapidly as the *second* generation.^4,7^ FDA-approved/listed platforms, such as CellSearch® (Silicon Biosystems) and ClearCell® FX (Biolidics) are exemplary technologies that have been widely used and demonstrated the clinical significance of CTCs.^8–12^

Currently, 265 clinical trials regarding CTCs are listed on clinicaltrials.gov. Despite successful CTC enumeration, achieving high yield and high purity remains challenging because of millions to billions of blood cells and a few to tens of CTCs present as background and target cells, respectively, in a milliliter of whole blood from cancer patient.^13^ It has been posited that the conventional EpCAM-based enrichment method would require 5 L of blood to detect at least one CTC in metastatic disease with 99% sensitivity.^9^ Such exceptionally low CTC frequencies could be attributed to progressively lost expression of epithelial markers during epithelial-to-mesenchymal transition (EMT) in circulation,^14,15^ as higher CTC counts have been reported with immunologic or physical property-based enrichment.^16–18^

In addition to the wide range of CTC detection rate reported in clinical studies, broad phenotypic plasticity and diversity have been observed at multiple molecular levels during metastatic cascade – from EMT and invasion^19–21^ to evasion of apoptosis,^22^ chemoresistance,^23^ migration,^24^ intravasation,^25^ extravasation, and organ colonization.^26^ While a tumor biopsy from either primary tumor or metastatic lesion alone may not always recapitulate the entire tumor harboring segregated clones,^27^ spatiotemporally heterogeneous CTCs collected in a sequential manner could potentially reveal comprehensive window into the metastatic disease for real-time monitoring of therapy response, which remains an unmet need in current clinical practice with tissue biopsy.

## Single-cell analysis

Emerging sequencing data from spatially distinct tumors provide clear evidence of intratumoral heterogeneity.^28–30^ Owing to the technical challenges, however, CTC analyses have been limited to bulk-cell samples, missing the information on cellular heterogeneity. The inevitable leukocyte contamination in any given primarily enriched sample further complicates downstream molecular analyses. Such confounding effect is particularly pronounced in transcriptomic studies when the activated leukocytes concurrently overexpress cancer-associated biomarkers, such as MUC1 or HER2, masking the true expression of CTC-specific transcripts.^31^ Their mesenchymal nature and hematopoietic origin further interfere with the expression of EMT-related and stem cell markers, respectively, resulting in false-positive observations.^32^

The transition from bulk to single-cell analyses on patient-derived CTCs has thus been fueled in part by studies over the past five years. At the genomic level, they have identified clinically relevant alterations, ranging from *small-scale* (e.g., single nucleotide variation (SNV), microsatellite instability) to *large-scale* mutations (e.g., copy-number variation, large-scale state transition, inter/intrachromosomal rearrangement). These aberrations include time-varying SNVs during the course of chemotherapy,^33^ private mutations that are absent in either matched primary or metastatic tumor^34^ and that are not yet listed in the COSMIC database (http://cancer.sanger.ac.uk),^35^ and copy-number profiles that distinguish chemosensitive from chemorefractory disease.^23^

Although limited in sample size and number of studies, transcriptomic studies have further revealed complex and heterogeneous expression patterns within and across patients. For example, expression profiles of single CTCs have demonstrated superior diagnostic accuracy in defining lineage identity and in identifying clinically distinct subsets of tumors across multiple myeloma and prostate cancers.^36,37^ They have also revealed therapeutically relevant biomarkers^38–40^ (e.g., predictive of resistance and/or response to adjuvant therapies), that are involved in activated oncogenic signaling pathways^41^ (e.g., PI3K-AKT-mTOR) and that are potentially targetable.^24,36,38,41–43^

## Integrated workflow

Despite the prevalence of EpCAM^−^ CTCs^44^ and varying capture efficiency,^45^ epithelial marker-dependent CellSearch® technology remains as the most common enrichment method to isolate CTCs from patient-derived peripheral blood. Pre-enrichment is often required for recovery of preferably viable and intact CTCs, and can be performed with direct imaging modalities,^36^ density gradient centrifugation in Percoll or Ficoll,^24,32^ immunoaffinity,^42,46–48^ microfiltration in two^43^ and three^41^ dimensions, and microfluidic approaches.^37–40,49–51^ Table S1 summarizes cell sorting and isolation technologies, including methods, working principles, features, limitations, and the reported recovery rates of spiked cancer cells. Primarily enriched bulk CTC samples are subsequently subjected to manual cell picking or micromanipulation,^24,32,36,41–43,46,48,49^ and micro/nanoplatforms^37–40,47,50,51^ for single-cell isolation and downstream molecular and phenotypic characterization (Table 1).

**Table 1:** Workflow summary of single-cell transcriptomic studies that analyzed patient-derived CTCs

Cancer type	CTC enrichment	CTC criteria (micromanipulation)	Single-cell profiling	Number of CTCs (number of patients)^a^	Reference
Multiple myeloma	FACS with serial dilution	CD45^−^, CD138^+^	SMART-seq2	21 (2)	^36^
Colon	CellSearch®	CD45^-^, EpCAM^+^	Multiplex PCR	11 (8)	^24^
Ovary	Biocoll separation, Dynabeads® CD45 depletion	DAPI^+^, CK/EpCAM^+^, CD45^-^	Multiplex PCR	15 (3)	^32^
Breast	MagSweeper®	EpCAM^+^	Microfluidic RT-PCR^b^	105 (50)	^42^
	Microfluidic ^neg^CTC-iChip	EpCAM/HER2/CDH11^+^, CD45/CD16/CD14^−^	Optimized Tang’s method	15 (10)	^49^
	Microfluidic CTC-iChip	EpCAM/HER2/EGFR^+^, CD45^−^	SMART-seq v4^c^	15 (10)	^38^
	Microfluidic ClearCell® FX	CD45/CD31^−^, Calcein^+ d^	Polaris^TM^ IFC	68 (4)	^50^
Melanoma	MagSweeper®	CD45^−^, Calcein^+^	SMART-seq	6 (1)	^48^
Prostate	MagSweeper®	CD45^-^, EpCAM^+^, DAPI^-^	SMART-seq, Advantage 2 PCR (Clontech)	20 (4)	^46^
	ScreenCell®	CD45^−^	Microfluidic RT-PCR^e^	38 (9)	^43^
	Microfluidic CTC-iChip	CD45^-^, EpCAM/CDH11^+^	Modified Tang’s method	77 (13)	^37^
Lung	Integrated nanoplatform	EpCAM^+^	Multiplex PCR	8 (1), 18 (1), 74 (1)	^47^
	Microfluidic ClearCell® FX	CD45^− f^	Multiplex PCR	61 (20)	^76^
Prostate, breast	CellSearch®, Parsortix^TM^	EpCAM/pan-keratins^+^	Multiplex PCR	13 (1), 8 (1)	^41^
Pancreas, breast, prostate	Microfluidic CTC-iChip	CD45^−^	Modified Tang’s method	7 (−), 29 (−), 77 (−)	^51^

^a^Number of CTCs (patients) included in the final analysis

^b^NanoFlex^TM^ 4-IFC Controller and BioMark^TM^ Real-Time PCR System

^c^Droplet digital PCR (Biorad ddPCR^TM^)

^d^Microfluidic Polaris^TM^ was used for single-CTC isolation

^e^BioMark^TM^ HD MX/HX system

^f^Microfluidic chip was used for single-CTC isolation

**Fig. 1:**
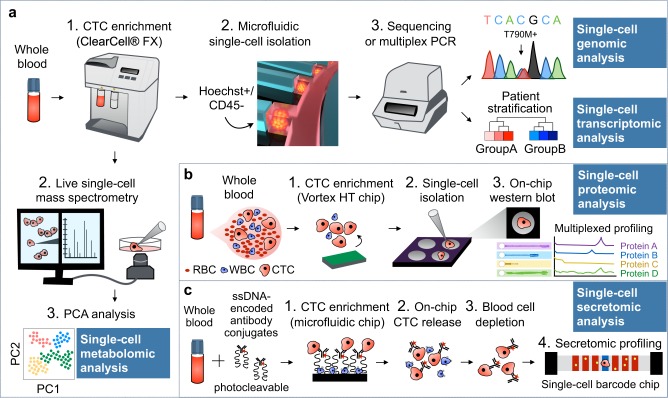
Microfluidic technologies for single-cell molecular characterization of patient-derived CTCs. **a** ClearCell® FX-integrated workflow. Single-cell genomic analysis: High concordance rate of EGFR mutations (T790M and L858R) was found between NSCLC CTCs and matched primary tumors.^12^ Single-cell transcriptomic analysis: Patient classification was done for breast cancer and NSCLC through full-length mRNA transcriptomic analysis^50^ and targeted gene expression profiling,^76^ respectively. Single-cell metabolomics analysis: Supervised principal component analysis (PCA) revealed unique metabolic profiles between CTCs and lymphocytes in gastric and colorectal cancer patients.^117^ **b** Single-cell proteomic analysis: Microfluidic single-cell western blotting (scWB) enabled the rapid analysis of an eight-plex protein expression in ER^+^ breast cancer.^60^ **c** Single-cell secretomic analysis: The integrated microfluidic on-chip system revealed highly heterogeneous expression profiles of two secreted proteins (i.e., IL-8 and VEGF) in CTCs from lung cancer patients.^118^

Microfluidics has particularly come to the fore in the field among the various isolating and cell sorting devices incorporating hydrodynamics,^12^ optics,^52^ dielectrophoresis,^53^ magnetics,^54^ or acoustics.^55^ The ability to manipulate even a small drop of whole blood and to retain cells or molecules at defined locations inside microfluidic devices enable characterization of chemical, thermal, and/or temporal variations in a multiplex manner. Having the benefits of integrated functionalities, microfluidic devices further eliminate the need for independent multiple modules required for sample preparation, purification and analysis, depending on input characteristics. Microfluidic technologies have increasingly been applied to rare cell populations, including CTCs, to explore multiple modalities of CTCs at the single-cell level (Fig. 1). These workflows coupled microfluidic systems, such as CTC-iChip,^37–40,51^ ^HB^CTC-Chip,^49^ ClearCell® FX,^12,50,56^ NanoVelcroChip^57,58^ and Parsortix,^41^ with single-cell profiling technologies, such as whole genome/exome sequencing (WGS/WES),^56,58^ single-cell RNA sequencing (scRNA-seq) or PCR (scPCR), single-cell western blotting (scWB) and secretion profiling.^59,60^

Gene-specific targeted preamplification or whole transcriptome amplification (WTA) is required prior to sequencing or profiling to analyze less than 1 pg of mRNA from the isolated single cells. Current WTA methods include PCR-based, multiple displacement amplification (MDA) or in vitro transcription (IVT)-based amplification^61^ of cDNA templates transcribed from single-cell mRNA. Many, however, are limited to selective amplification of the polyadenylated RNAs, and thus may be biased to the 3’-end or the 5’-end of a transcript.^62^ Among a few WTA techniques developed for full-length mRNA-characterization of a single cell, modified versions of SMARTer^48^ (e.g., SMART-Seq2) and that of Tang’s method^63^ are commonly employed in single-CTC transcriptomic studies aiming to achieve improved transcript detection, coverage, accuracy, and yield (Table 1).

For quantitative transcriptomic analysis, an accurate identification of technical artifacts from intrinsic biological cellular variability is critical to prevent spurious readings from single CTCs. Ideally, quality control (QC) metrics should be performed with the amplified cDNA products after preamplification step, given the amount of genetic material minimally required. Current single-cell transcriptomic studies on patient-derived CTCs have assessed (1) the yield or concentration of amplified DNA,^36,64–66^ (2) Cq values for selected reference, or *housekeeping*, genes using qPCR,^33,58^ (3) fragment size distribution of selected DNA sequences using gel/capillary electrophoresis,^36,58,64,67–71^ and (4) genome integrity index (GII), which ranges from 0 (poor quality) to 4 (high quality) and is computed based on PCR bands of four primer pairs using gel electrophoresis.^23,72,73^

Cells harboring QC-passed RNAs are subsequently subjected to library construction, followed by scRNA-seq^36,38,40,46,48–51^ or quantitative profiling with conventional qPCR,^24,32^ digital droplet PCR (ddPCR)^39,40^ or microfluidic dynamic array.^42,43^ The quality of constructed libraries are further validated with (1) the proportion of reads mapping to genome, and/or (2) the number of genes detected.^50^ Lineage specificity of CTCs is often confirmed by high expression of cancer-specific markers and low expression of leukocyte markers with pre-specified thresholds.^37^ Low success rate of <60% for overall amplification and library preparation attributed to multiple processing steps has been reported in CTC studies, highlighting the need to systematically quantify QC metrics prior to the analysis.

## Expression data

Single-cell transcriptome of patient-derived CTCs have been analyzed comparatively with cancer cell lines, white blood cells (WBCs), matched primary tumors and/or metastases.^24,37,42,43,48^ Alternatively, expression levels were assessed and compared between CTC subgroups defined by unsupervised hierarchical clustering or other classification methods.^41,49^ In the following sections, we focus on the most relevant gene signatures that are perceived to be critical determinants of metastasis and disease outcome and that are commonly differentially expressed in CTCs at the single-cell level: EMT, stemness, interaction with blood components, DNA repair, signaling pathways and drug targets (Table 2).

**Table 2:** CTC phenotypes and the related gene signatures expressed in patient-derived single CTCs

Significance	Gene signatures	Cancer type	Reference
Epithelial	EpCAM, KRT5, KRT7, MUC1	Ovary	^32^
	EpCAM, KRT8, KRT18, KRT19, CTNNB1	Breast	^42^
	EpCAM	Breast	^49^
	EpCAM, KRT7, KRT8, KRT18, KRT19	Prostate	^37^
	EpCAM, KRT19	Prostate	^41^
	EpCAM, KRT18, KRT19, CEACAM7	Colon	^24^
Mesenchymal/EMT	CDH2, VIM, SNAI2, CD117, CD146	Ovary	^32^
	TGFB1, FOXC1, CXCR4, NFKB1, VIM, ZEB2	Breast	^42^
	CDH2, MMPs, PTPRC, VIM, ZEB1, ZEB2	Prostate	^41^
	S100A9, NPTN, S100A4	Breast	^42^
	CDH11	Breast	^49^
	CDH2, CDH11, FN1, VIM, SERPINE1	Prostate	^37^
	IGF1, IGF2, EGFR, FOXP3, TGFB3, PTPRN2, ALDH1, ESR2, WNT5A	Prostate	^43^
	VIM	Colon	^24^
	FN1, CD44v6, CD151, TSPAN8	Ovary	^32^
Stemness	CD44, ALDH1A1, NANOG, OCT4	Ovary	^32^
	CD44, ALDH7A1, KLF4	Prostate	^37^
	CD24, CD44	Breast	^42^
	CD24, CD44	Prostate	^41^
	CD44, PTEN, CD133, NKX3-1, MYC, ATXN1, GATA3, TNFSF11, TNFRSF11B, TACSTD2	Prostate	^43^
	CD166, CD26, CD44s	Colon	^24^
DNA repair	PARP1	Breast	^42^
	RAD51	Prostate	^41^
Interaction with platelets	ITGA2B, ITGB3, SELP	Breast	^49^
Immune-related	CXCL14	Breast	^38^
	CCL4, CXCL2, CXCL9, IL15, IL1B, IL8	Prostate	^41^
	CD47, CALR	Colon	^24^
	HLA-G, HLA-H, HLA-C, HLA-B, TRPM1	Melanoma	^48^
Signaling pathways, drug targets	SERPINA3, WFDC2, FAT1, FAT2, SFRP1, SFRP2	Breast	^38^
	AKT1, AKT2, PIK3R1, PTEN	Breast	^42^
	EGFR, HER2	Breast	^49^
	Hormone signaling (AR non-genotropic, GR), growth factor signaling (MET, ERBB1 downstream, SMAD2/3 nuclear, SMAD2/3, TGFBR), cell adhesion signaling (Nectin, EPHA2 fwd, E-cadherin stabilization, E-cadherin nascent AJ)	Prostate	^37^
	AR, AR-V7, ERBB2, EGFR, PIK3CA, MTOR	Prostate	^41^
	Drug targets (PIM3, MTOR, ACP5, PIM1, PIM2, AXL, ALPL, SPP1, ADRA2A, HERPUD1, AURKA, MUC1), Wnt signaling, SHH signaling, TGFB signaling, EGFR signaling	Prostate	^43^
	CD38, SLAMF7, BCMA	Multiple myeloma	^36^

In line with molecular evidences supporting EMT-driven metastasis,^24,32,41–43,49^ bulk-cell studies have suggested the contribution of EMT to early steps of the metastatic spread (i.e., tumor invasion, intravasation, CTC generation and survival, and early seeding in secondary organs).^74^ Nonexclusive hypotheses of EMT’s contribution to CTC biology suggest that (1) CTCs may have been mesenchymally-shifted in primary tumors to have enhanced survival properties through activation of genes involved in survival pathways and escape from immune surveillance, or (2) undergo EMT processes within the bloodstream by means of TGFß liberated from circulating platelets.^74^ At the center of the research axis is to identify and characterize such premetastatic subsets of CTC population that are favored to be liberated from primary tumors and survive in the bloodstream to succeed in the early colonization phases. scRNA-seq is particularly well suited in this regard to discover distinct subsets of CTCs capable of forming metastasis.

Emerging single-cell profiling data provide clear evidence of a continuum in the development of CTC phenotypes, including epithelial (E), epithelial-mesenchymal (E/M), mesenchymal (M) and stem-like phenotype. Highly heterogeneous expression of epithelial markers (e.g., EpCAM, CK18, CK19) was observed in CTCs across colorectal,^24^ ovarian,^32^ breast,^42^ and prostate^37,41,43^ cancers. Similarly, mesenchymal or EMT-related genes (e.g., CDH2, VIM, TGFß1, ZEB1/2) were commonly enriched in single ovarian,^32^ breast,^42^ and prostate^41,43^ CTCs. Compared to matched primary/metastatic tumor tissue and cell lines, migration-related and cell-cell adhesion genes (e.g., TSPAN8, CD151, CD44v6, and FN1) were generally downregulated in colon^24^ and prostate CTCs,^43^ respectively, possibly suggesting a lack of their need for mobility and migratory capabilities in the bloodstream in these cancer types.

While some studies have suggested that CTCs that are ‘frozen’ in either E or M state lacking EMT plasticity are unable to form metastases,^75^ the repetitive observation of patient-derived CTCs expressing mesenchymal attributes directly correlated with the appearance of metastases in recent studies suggest that these mesenchymal-shifted cells, and not *benign* cells passively detached from a primary tumor, are precursors of metastasis.^76^ Clinical data linking CTC-derived EMT markers with multiple clinical parameters are discussed elsewhere.^74^ It remains to be elucidated whether there exist specific hybrid E/M states that are particularly prone to perturbations triggering extensive phenotypic and functional change in circulation. Computational analytical tools enabling pseudo-time reconstruction of transitioning cells^77–79^ and the topography underlying E/M plasticity^80^ from a static single-cell gene expression data may be applied to prospective studies to clarify the nature of hybrid E/M states and define their role in metastasis.

It has been suggested that tumor cells having an intermediate phenotype of EMT show the highest plasticity and thus represent cancer stem cells (CSCs).^81^ Varying expression levels of stem cell markers (e.g., CD24, CD44, ALDH1A1, NANOG, and OCT4) were found at the single-cell level in ovarian,^32^ breast^42^, and prostate CTCs.^37,41^ Interestingly, genes involved in oncogenic signaling pathway were found to be differentially expressed in single CTCs depending on the level of cellular plasticity or stemness. In prostate cancer, for example, expression of the key regulators in the PI3K/Akt/mTOR signaling pathway (i.e., PI3K, mTOR) were highly expressed in CD44^−^/CD24^+^ CTCs.^41^ This subset of CTC populations may thus be more susceptible to perifosine (Akt inhibitors) and rapamycin (mTOR inhibitors) treated with conventional chemotherapy or radiotherapy.^82^ Given that both CD44^+^/CD24^−^ and CD44^+^/CD24^+^ tumor cells have functional significance in initiating tumor growth^83^ and that CTCs express these two cell surface markers across various malignancies, it remains to be investigated whether their expression has comparable functional significance in circulation.

The innate immune regulator, CD47, was the only gene that was upregulated in CTCs compared to matched tumor tissue in colorectal cancer,^24^ suggesting a potential immune-escape mechanism associated with CTC survival in circulation. Another form of immune evasion was suggested in melanoma, where the genes associated with the escape from immune surveillance, including HLA genes (i.e., HLA-G, HLA-H, HLA-C, and HLA-B) and TRPM1, were significantly downregulated in CTCs compared to melanoma cell lines, primary melanocytes, human embryonic stem cells, and lymphoma cell lines.^48^ Transcriptional repression of HLA genes has been associated with complete loss of MHC class I membrane expression, and importantly, the primary resistance to immune checkpoint inhibitor (ICI)-based immunotherapy.^84^ The screening of plasma-membrane proteins through whole transcriptomic analysis is thus of utmost interest to identify not only CTC-specific diagnostic biomarkers^48^ but also immune escape and survival mechanisms underlying resistance to immunotherapy.

Another key player is TGFß-releasing platelet, which may adhere to CTCs in the bloodstream.^19,85^ In line with this hypothesis, platelet markers were frequently expressed in isolated single CTCs, as well as in CTC clusters in breast cancer.^49^ Labelle et al. showed that TGFß liberated from platelets may induce EMT in tumor cells within the bloodstream and further promote the formation of the early metastatic niche.^85,86^ Based on these observations, it is speculated that TGFß expressing CTCs may represent a specific subpopulation having high metastatic potential. Importantly, platelet-dependent natural killer (NK) cell escape mechanism has been suggested by in vitro and preclinical models across diverse mouse and human cancer cell lines.^74^ It is thus posited that the presence of platelets may equip tumor cells with enhanced ability to escape elimination by the immune system through EMT, ultimately promoting their metastatic competency.

Genes involved in DNA repair (e.g., RAD51, PARP1) and G2/M DNA damage checkpoint (e.g., AR, TK1, PLK1, MAGEA1, MAGEC1, MAGEC2, CTAGB1, BIRC5, TOP2A) were frequently expressed in prostate CTCs.^41,46^ While several transcripts (e.g., PLK1, TOP2A) have been associated with aggressiveness in localized prostate cancer,^46^ it is noteworthy that CTCs derived from advanced cancer patients also highly expressed these markers relative to normal prostate tissues. In contrast, compared to cancer cell lines and matched primary tumors, genes involved in cell proliferation (e.g., MYC, ATF3, TERT, RAC1, FOXA1, RRM1, CCNB1, BIRC5, Ki-67, c-Myc) were significantly downregulated in CTCs across breast^42^ and colorectal^24^ cancers, suggesting a non-proliferative, or *dormant*, state of CTCs in circulation.

Given the generally diminished expression of proliferation-related genes, conventional therapeutic strategies targeting proliferating cells may not be the best for eradicating “seeds” of metastasis. Promisingly genes involved in the PI3K-AKT-mTOR signaling pathway, in which many are currently in (pre)clinical trial stages or FDA-approved, and other potentially targetable genes were frequently expressed in CTCs at high levels across various malignancies including multiple myeloma,^36^ and breast,^38,42^ prostate^41,43^ and colorectal^24^ cancers. The incorporation of single-CTC analysis into clinical trials may thus be ideal from clinical perspective for the development of companion diagnostics.^42^

## Clinical significance

scRNA-seq or scPCR technologies have been widely applied to study early mammalian development, neuronal diversity, and immune system, revealing spatial and temporal dynamics, cellular heterogeneity, clonal distribution, pathways, and crosstalk.^87–89^ Their application in the context of CTC-based liquid biopsy, however, has been limited primarily to capturing a snapshot of the cellular states at a given point in time. In clinical contexts, it is the *dynamics* of such cellular state (i.e., temporal heterogeneity) that is of primary interest to monitor therapeutic response during the course of treatment. Despite technical challenges, a few single-cell studies have successfully demonstrated clinically-promising use of CTC-derived transcripts particularly for serial monitoring of the disease in a prospective cohort.

Prior knowledge of genes of interest is often required in traditional gene expression analysis for clinical diagnosis of tissues or cells in circulation using immunohistochemistry, in situ hybridization, or flow cytometry, yielding semi-quantitative data. In contrast, scRNA-seq generates high-throughput expression data in an unbiased, objective manner, with superior diagnostic sensitivity over existing technologies. For example, scRNA-seq-acquired expression data of a few selected, well-established markers, which were previously used to sort multiple myeloma (MM) cells by flow cytometry, achieved near perfect accuracy in differentiating normal and malignant plasma cell.^36^ Similarly, the improved diagnostic performance of CTC-based multiplex assays was observed in advanced breast cancer, supporting the robust detection capability of single-CTC-derived markers.^38^

The benefits of scRNA-seq technologies in the CTC field are particularly pronounced in classifying sub-populations of cells that may be clinically distinct, which are overlooked by conventional diagnostics due to the insufficient resolution. Unsupervised hierarchical clustering of single circulating MM cells-derived transcripts, for example, differed considerably from one patient to another, indicating the presence of different subtypes in MM.^36^ The existence of key chromosomal translocations associated with clinical risk may further be inferred from scRNA-seq data; circulating MM cells overexpressed CCND1 and CCND3 indicative of chromosomal translocations of CCND1/IGH fusion from t(11;14) and CCND3/IGH fusion from t(6;14)), respectively, and the presence of these genomic aberrations were further validated in matched MM by fluorescence in situ hybridization (FISH).^36^ Provided that the overexpression of CCND1 has been associated with resistance to EGFR-, BRAF- and MAF-targeted therapies,^90^ single-CTC transcriptome may be used as a predictive indicator for diagnosis, MM classification, and therapeutic efficacy in clinical settings.

Transcriptomes of single CTCs have been analyzed comparatively with that of CTC clusters, which have been associated with enhanced metastatic competence^91–93^ and poor prognosis^49,94,95^ across multiple cancer types. Differential expression analysis between the two groups identified specific gene signature (e.g., cell junction component plakoglobin) required in forming CTC cluster and distant metastases, in which high expression levels were indicative of short metastasis-free survival.^49^ Further, single-CTC-derived transcriptomes revealed signaling pathways (i.e., non-canonical Wnt signaling) relevant to treatment response (i.e., anti-androgen resistance), which was not evident in matched primary tumors, in prostate cancer.^37^ Similarly, the potential role of Sonic Hedgehog, Wnt, and TGFß signaling pathways in metastatic castration-resistance and immunotherapy response was suggested by single-CTC expression profiles in prostate cancer.^43^

By applying label-free microfluidic approaches,^12^ our group recently demonstrated how previously developed prognostic index,^96,97^ and the resulting prognostication, can be refined with single-CTC-derived gene signatures while accounting for cellular heterogeneity.^76^ Expression of a subset of matrisome genes, including MMP1 and MMP12, in tumor tissues and CTCs was consistently associated with metastatic spread and early recurrence of non-small-cell lung cancer (NSCLC), respectively. In line with our earlier observations of EGFR mutations (e.g., T790M/L858R) in CTCs and matched tumor tissue,^12^ this study provides the molecular evidence linking a sold tumor with single-CTC-based indicator associated with clinical presentation at the transcript level, highlighting promising predictive value of circulating biomarkers.

Despite a well-established role as a biomarker prognostic of survival particularly in breast cancer,^98,99^ microscopy-based CTC enumeration alone may not be sufficient to predict drug resistance in the absence of robust molecular characterization. As an advanced alternative, single-CTC-derived transcripts may serve as an excellent source to develop quantitative scoring assay comprising tissue lineage-specific genes that are not present in normal blood cells. To date, very few studies have applied such metrics in a prospectively monitored patient cohort to demonstrate their practical utility in the clinical setting.^38–40^

In 2018, the Haber group presented a predictive digital CTC scoring strategy to identify patients with poor overall survival (OS) and progression-free survival (PFS) in metastatic castration-resistant prostate cancer treated with first-line abiraterone.^40^ Serial monitoring of CTCs further predicted early dissemination in another independent cohort of patients with localized cancer.^40^ The same group found that digital quantification of intracellular ER signaling in single CTCs was predictive of residual disease in localized breast cancer patients treated with neoadjuvant therapy.^38^ Importantly, this 17-gene CTC score predicted early progression in metastatic breast cancer treated with endocrine therapy, which was not adequate to suppress ER signaling, despite having functional ESR1.^38^

The greatest focus in immuno-oncology has been on tumor biopsy-derived features, such as PD-L1 expression, tumour-infiltrating lymphocyte (TIL) density, T-cell receptor (TCR) clonality, mutational burden, and immune gene signatures, for their increasingly recognized predictive values for ICI-based immunotherapy.^100^ Although promising, their invasive nature makes repeated sampling not clinically practical particularly for metastatic diseases over the course of treatment. The development of less-invasive CTC-based liquid biopsies as a predictive biomarkers for response to ICI treatments will therefore be particularly promising. Hong et al. recently showed that the scoring model recapitulating temporal dynamics of CTCs identified patients with better OS and PFS in ICI-treated melanoma patients, demonstrating the feasibility of quantifying transcripts derived from microfluidically enriched CTCs for predicting patients likely to benefit from ICI therapies.^39^ Larger studies will be required to develop and establish such generalized framework for guiding therapeutic decision-making.

## Challenges and beyond

The advent of sequencing technologies has created a new era of precision medicine. The prospect of applying this concept to develop clinically applicable biomarkers for diagnosis, prognosis, and prediction of therapy response has been extensively explored on cancer patients. Particularly, liquid biopsies focusing on the analysis of CTCs and cell-free tumor DNA (ctDNA) in the bloodstream are evolving into promising clinical parameters.^101^ ctDNA may allow mutational analyses to monitor tumor dynamics during cancer treatment^102^ and offer easier handling, storage, and shipping of samples compared to CTCs.^103^ A small number of mutant gene fragments present in ctDNA, which are further diluted by normal circulating DNA fragments released by apoptotic cells, however, require highly sophisticated methods to accurately assess tumor-specific genomic alterations (a detailed comparison between the two types of analytes is beyond the scope of this review). Nevertheless, a recently developed CancerSEEK blood test that examines the presence of mutations in cfDNA has achieved high sensitivity ranging from 69 to 98% across five cancer types, showing great promise for early cancer detection.^104^

CTCs represent intact and viable tumor cells that can be analyzed at multiple biological levels, allowing sequential sampling at multiple time points from patients undergoing systemic drug treatment. It is thus possible to perform multi-dimensional molecular and phenotypic characterization of these cells, which increasingly serves as an essential tool in precision diagnosis.^105^ Nevertheless, challenges remain in the field as these putative metastatic precursor cells occur at extremely low frequency relative to normal leukocytes in any given clinical sample. Such rare nature of CTCs clearly raises the question of whether these cells obtained at a single time point alone would truly recapitulate spatially and temporally evolving landscape of the entire tumor and its microenvironment, or the metastatic state. Further, the recovery efficiency varies greatly across the enrichment technology, posing additional challenges in understanding their cellular heterogeneity and the functional and clinical significance of their appearance in the bloodstream. Consequently, little is known about the molecular characteristics and mechanisms, particularly in relation to drug resistance and their capacity in circulating bloodstream with metastatic potential.

The confounding effects of inherent rarity and heterogeneity of CTCs on the downstream analysis may further be exacerbated by biased positive selection during single-cell isolation (i.e., enrichment of target cells based on antibodies specific to CTC surface markers), missing out cells with low or even no surface marker expression in circulation which prove to be of clinical significance.^7^ CTCs in advanced disease indeed exhibited predominant epithelial-mesenchymal-mixed (E/M), or mesenchymal (M) phenotypes (i.e., expressing mesenchymal markers) across multiple cancer types, including esophageal squamous carcinoma,^106^ ovarian cancer,^107^ pancreatic cancer,^108^ colorectal cancer,^109^ triple-negative breast cancer,^110^ and hepatocellular carcinoma.^111^ These EMT-shifted CTCs would not have been detected by immunoaffinity-based enrichment solely facilitated by antibodies targeting epithelial markers (e.g., EpCAM and pan-keratins). Capture efficiency may thus be enhanced by using cocktails of antibodies,^7^ including both epithelial and mesenchymal biomarkers, or by utilizing tumor lineage-specific signatures^38^ without making a priori assumption about the type of tumor cells.

In contrast, negative depletion (i.e., removal of non-target cells) using label-free approaches which leverage unique physical properties (e.g., cell size) of CTCs may lead to relatively low purity given the size overlap with leukocytes,^112^ as observed across breast, colorectal and prostate cancers.^113^ Some may even present a similar immunofluorescence staining pattern with leukocytes expressing both leukocyte- and CTC-specific markers, adding layers of complexity. Although such “double positive” cells are often excluded from the analysis, their occurrence in healthy blood samples at a much lower frequency point towards their possible functional role and clinical impact.^114^ Microfluidic approaches are increasingly being applied in this regard to enable both WBC elimination and selective CTC isolation on a single platform, as demonstrated by two-stage microfluidic chips.^115,116^

Finally, many single-CTC studies do not state the total number of cells initially isolated during the enrichment step, and the quality and number of CTCs that have failed QC and that have been excluded from further analysis. This makes the direct comparison of transcriptional changes found in CTCs between studies extremely difficult, as such molecular findings may only be applicable to a small subpopulation of CTCs depending on the enrichment technology or the QC metrics. The development of a clearly defined and more uniform workflow is thus urgently needed to facilitate its clinical application at different stages of the antitumor therapy or cancer progression across, and within, patients. The 17-gene CTC-specific assay is an exemplary quantitative scoring metrics that has achieved high sensitivity for monitoring of therapy response in localized and metastatic breast cancer patients.^38^ Continuous optimization of the developed platform and prospective clinical validation of CTC-based liquid biopsy will ultimately provide clinicians with robust, yet readily understandable, test results in a shorter turnaround time compared to conventional tissue biopsy.

## Supplementary information

Supplementary Information
