# Supplementary material for: Liquid biopsy: one cell at a time
Source: NPJ Precis Oncol. 2019 Oct 2;3:23. doi: 10.1038/s41698-019-0095-0 (PMC6775080; doi:10.1038/s41698-019-0095-0)
Supplement: Supplementary file 1 — Supplementary Information [file 41698_2019_95_MOESM1_ESM.pdf]

Supplementary Information (SI) for

Liquid biopsy: one cell at a time

Su Bin Lim, Wen Di Lee, Jyothsna Vasudevan, Wan-Teck Lim, Chwee Teck Lim

Corresponding author: Chwee Teck Lim
Email: ctlim@nus.edu.sg

This PDF file includes:

Table S1
SI References

Supplementary Tables

Table S1. CTC enrichment technologies.

Method	Working principle	Features and limitations	Technology	Isolation efficiency	
				Cell lines spiked into healthy blood	Recovery rate (%)
Density gradient centrifugation[1,2]:	Separation based on the migration of cells through a medium of higher or graded density, which is dependent on their sedimentation coefficient, when the cells are exposed to an elevated centrifugal force[3,4]	• Low cost<br>• Ease of use<br>• Label-free<br>• Low yield and low purity due to formation of aggregates[64]<br>• Additional enrichment techniques required[64]	Ficoll-Paque and negative enrichment using Dynabeads® CD45	CAL54, CAKI-1, CAKI-2, A498	32-77[5]
			Ficoll-Paque and negative enrichment using RosetteSep™	CAL54, CAKI-1, CAKI-2, A498	7-53[5]
			OncoQuick®	MDA-361	55.2[6]
			AccuCyte® - CyteFinder®	LnCAP, A549, MCF7, PC3	90.5[7]
Microfiltration in two[8] and three[9] dimensions	Sample filtration through an array of microscale constrictions and isolation of CTCs based on both cell size and deformability[3,10,11].	• High throughput[3,12]<br>• Low cost[13]<br>• Ease of use[11]<br>• High cell viability and quality<br>• Low purity and low specificity<br>• False-negatives (CTCs smaller than pore size) and false-positives (large blood cells)[12,13]<br>• Size can be affected by cell deformability and stiffness<br>• Membrane clogging<br>• Difficult to detach CTCs from filter	ScreenCell®	H2030	74-91[14]
			ISET®	MCF7, SKBR3, MDA-MB-231	83-100[15]
			CellSieve™	MCF7	~90[16]
			Flexible MicroSpring Array (FMSA)	MCF7, MDA-MB-231, C8161, WM35	~90[17]
			FaCTChecker	MCF7, MDA-MB-231	78-83[18]
			Parsortix™	CAL54, CAKI-1, CAKI-2, A498	30-87[5]
				T24, PANC-2	42-70[19]
			SmartBiopsy™	H358	82-87[20]
Inertial microfluidics	Separation based on the position of cells in a flow channel, which size dependent[21].	• High throughput<br>• Label-free[22]<br>• High cell viability<br>• Easy of use<br>• False-negatives and false-positives[64]	ClearCell® FX	T24, MCF7, MDA-MB-231	80-87[23]
			Vortex chip	MCF7	20.7[24]
					>83[25]
Dielectrophoresis (DEP)	Separation of cells based on their electrical properties by generating a non-uniform alternating current field in the *microchips*[64].	• Single-cell resolution<br>• High cell viability<br>• High throughput[3,12]<br>• Pre-enrichment step required<br>• Low purity	ApoStream™ system	A549, ASPS-1, MDA-MB-231	64.9-69.9[26]
			DEP and field-flow fractionation (FFF)	MDA-435	10-92[27]
					75[28]

	However, electrical properties of CTCs are not fully understood and validated as differentiating criteria between CTCs and leukocytes[11]	• Gradually changing dielectric characteristics of CTCs due to ion leakage<br>• Low electric conductivity of the medium required[64]			
Acoustophoresis	Separation based on cells' acoustophoretic mobility, which is size dependent[21].	• High throughput<br>• High cell viability<br>• No prior enrichment required[29]<br>• High separation efficiency can be achieved with modification of acoustophysical properties of buffer[30]<br>• Recovery efficiency dependent on blood concentration and sample volume[30]	Bulk acoustic standing wave	DU145	83.7[31]
			Surface acoustic standing wave	MCF7, UACC903M, LNCaP	84-90[32]
			Bulk acoustic standing wave and DEP chip	DU145	~76[33]
Direct imaging modalities[34]	Technologies integrating microscopy and flow cytometry for identification of specific subpopulations of cells[21].	• High throughput data acquisition<br>• Real-time fluorescence intensity<br>• High-resolution multi-mode images of individual cells<br>• Cellular subpopulation analysis based on photometric and morphometric parameters[35]<br>• Multiplex immunophenotyping, FISH, PCR and SNP analysis<br>• Time consuming<br>• Limited depth-of-field images[35]<br>• Not fully automated for identification of CTCs<br>• False-positives and false-negatives<br>• Scattering and autofluorescence	CytoTrack™	MCF7	55-78[36]
			CTCScope™	MDA-MB-468	~71 [37]
			Epic science CTC platform	COLO-205	88[38]
			Photoacoustic flow cytometry (PAFC)	MDA-MB-231	96[39]

		background			
Functional assays	Enrichment based on the analysis of the bioactivity of viable cells[21].	• High sensitivity[13]<br>• Continuous active protein secretion by viable cells required<br>• Technical knowledge for maintaining cell culture required<br>• Time consuming[13]	EPISPOT	MCF7	37-100[40]
			Vita-Assay™	PC3	50 (CAM), 81 (MNC-CAM)[41]
Immunoaffinity[42-45]	Positive (i.e., CTCs) or negative (i.e., blood cells) enrichment based on highly-specific affinity between selected antibodies and target antigens.	• Relatively high sensitivity<br>• Varying capture efficiency due to heterogeneity and lack of robust biomarkers[3,11]<br>• Possible cytotoxic effect on CTCs due to antibody binding[22]<br>• False-positives (EpCAM/CK^+ circulating normal epithelial cells[3], circulating endothelial cells[46], activated leukocytes[4]) and false-negatives (metastatic, EMT-shifted CTC subpopulations[3,22])	Positive enrichment of CTCs		
			CellSearch® system	MCF7, SKBR3, MDA-MB-231	36.4% - 79%[15]
				SKBR3	>85[47]
			Dynabeads® Epithelial Enrich	A498, CAL54, CAKI-1, CAKI-2	0% - 60%[5]
			AdnaTest®	SKBR3	~100[48]
			MACS® CD326 (EpCAM) MicroBeads	MOR/P	75[49]
			MagSweeper®	MCF7	62[50]
			GEDI	Capan-1, PANC-1, BxPC-3	~30-60[51]
			OncoCEE™	SKOV	>70[52]
			Herringbone chip (HB-Chip)	PC3	91.8[53]
			IsoFlux™	PC3, MDA-MB-231	73-81[54]
			CTC-Chip[38,48-50,52]	H1650	>60[55]
			NanoVelcroChip[59,60]	MCF7	>95[56]
			GEM	L3.6pl, BxPC-3	>90[57]
			Graphene oxide (GO) chip	MCF7	73-94.2[58]
			Biofluidica CTC detection system	MCF7	83.1[59]
			Ephesia	MCF7	>90[60]
			Magnetic Sifter	LNCaP, MCF7, H1650, HCC827	>90[61]
			LiquidBiopsy®	MCF7	70-78%[62]
		• Time effective	Negative enrichment of leukocytes		

		• False-positives<br>• Contaminating leukocytes	EasySep™	SW620	58[63]
			MACS® CD45 MicroBeads	DU-145	70-88[64]
			Dynabeads® CD45	PM1	87.4[65]
			CanPatrol™	NCI-H2228	80-88[66]

SI References

1 Blassl, C. *et al.* Gene expression profiling of single circulating tumor cells in ovarian cancer - Establishment of a multi-marker gene panel. *Mol Oncol* **10**, 1030-1042, doi:10.1016/j.molonc.2016.04.002 (2016).

2 Steinert, G. *et al.* Immune escape and survival mechanisms in circulating tumor cells of colorectal cancer. *Cancer research* **74**, 1694-1704, doi:10.1158/0008-5472.CAN-13-1885 (2014).

3 Gabriel, M. T., Calleja, L. R., Chalopin, A., Ory, B. & Heymann, D. Circulating Tumor Cells: A Review of Non-EpCAM-Based Approaches for Cell Enrichment and Isolation. *Clin Chem* **62**, 571-581, doi:10.1373/clinchem.2015.249706 (2016).

4 Heymann, D. & Téllez-Gabriel, M. Circulating tumor cells: The importance of single cell analysis. in *Single Cell Biomedicine* (eds. Gu, J. & Wang, X.) 45-58 (Springer, Singapore, 2018).

5 Maertens, Y. *et al.* Comparison of isolation platforms for detection of circulating renal cell carcinoma cells. *Oncotarget* **8**, 87710-87717, doi:10.18632/oncotarget.21197 (2017).

6 Baker, M. K. *et al.* Molecular detection of breast cancer cells in the peripheral blood of advanced-stage breast cancer patients using multimarker real-time reverse transcription-polymerase chain reaction and a novel porous barrier density gradient centrifugation technology. *Clin Cancer Res* **9**, 4865-4871 (2003).

7 Campton, D. E. *et al.* High-recovery visual identification and single-cell retrieval of circulating tumor cells for genomic analysis using a dual-technology platform integrated with automated immunofluorescence staining. *BMC cancer* **15**, 360, doi:10.1186/s12885-015-1383-x (2015).

8 Chen, C. L. *et al.* Single-cell analysis of circulating tumor cells identifies cumulative expression patterns of EMT-related genes in metastatic prostate cancer. *Prostate* **73**, 813-826, doi:10.1002/pros.22625 (2013).

9 Gorges, T. M. *et al.* Accession of Tumor Heterogeneity by Multiplex Transcriptome Profiling of Single Circulating Tumor Cells. *Clinical chemistry* **62**, 1504-1515, doi:10.1373/clinchem.2016.260299 (2016).

10 Pugia, M., Magbanua, M. J. M. & Park, J. W. Enrichment and detection of circulating tumor cells and other rare cell populations by microfluidic filtration. in *Isolation and*

Molecular Characterization of Circulating Tumor Cells (eds. Magbanua, M. J. M. & Park, J. W.) 119-131 (Springer, Cham, 2017).

11 Hao, S.-J., Wan, Y., Xia, Y.-Q., Zou, X. & Zheng, S.-Y. Size-based separation methods of circulating tumor cells. *Advanced drug delivery reviews* **125**, 3-20 (2018).

12 Dolfus, C., Piton, N., Toure, E. & Sabourin, J. C. Circulating tumor cell isolation: the assets of filtration methods with polycarbonate track-etched filters. *Chin J Cancer Res* **27**, 479-487, doi:10.3978/j.issn.1000-9604.2015.09.01 (2015).

13 Alunni-Fabbroni, M. & Sandri, M. T. Circulating tumour cells in clinical practice: Methods of detection and possible characterization. *Methods* **50**, 289-297, doi:10.1016/j.ymeth.2010.01.027 (2010).

14 Desitter, I. *et al.* A new device for rapid isolation by size and characterization of rare circulating tumor cells. *Anticancer research* **31**, 427-441 (2011).

15 Kallergi, G., Politaki, E., Alkahtani, S., Stournaras, C. & Georgoulias, V. Evaluation of Isolation Methods for Circulating Tumor Cells (CTCs). *Cellular physiology and biochemistry : international journal of experimental cellular physiology, biochemistry, and pharmacology* **40**, 411-419, doi:10.1159/000452556 (2016).

16 Adams, D. L. *et al.* Precision microfilters as an all in one system for multiplex analysis of circulating tumor cells. *RSC Advances* **6**, 6405-6414, doi:10.1039/C5RA21524B (2016).

17 Harouaka, R. A. *et al.* Flexible micro spring array device for high-throughput enrichment of viable circulating tumor cells. *Clinical chemistry* **60**, 323-333, doi:10.1373/clinchem.2013.206805 (2014).

18 Zhou, M. D. *et al.* Separable bilayer microfiltration device for viable label-free enrichment of circulating tumour cells. *Scientific reports* **4**, 7392, doi:10.1038/srep07392 (2014).

19 Hvichia, G. E. *et al.* A novel microfluidic platform for size and deformability based separation and the subsequent molecular characterization of viable circulating tumor cells. *International journal of cancer* **138**, 2894-2904, doi:10.1002/ijc.30007 (2016).

20 Kim, E. H. *et al.* Enrichment of cancer cells from whole blood using a microfabricated porous filter. *Analytical Biochemistry* **440**, 114-116, doi:https://doi.org/10.1016/j.ab.2013.05.016 (2013).

21 Ferreira, M. M., Ramani, V. C. & Jeffrey, S. S. Circulating tumor cell technologies. *Molecular Oncology* **10**, 374-394, doi:10.1016/j.molonc.2016.01.007 (2016).

22 Hou, H. W. *et al*. Isolation and retrieval of circulating tumor cells using centrifugal forces. *Sci Rep* **3**, 1259, doi:10.1038/srep01259 (2013).

23 Warkiani, M. E. *et al*. Slanted spiral microfluidics for the ultra-fast, label-free isolation of circulating tumor cells. *Lab on a chip* **14**, 128-137, doi:10.1039/c3lc50617g (2014).

24 Sollier, E. *et al*. Size-selective collection of circulating tumor cells using Vortex technology. *Lab on a chip* **14**, 63-77, doi:10.1039/C3LC50689D (2014).

25 Che, J. *et al*. Classification of large circulating tumor cells isolated with ultra-high throughput microfluidic Vortex technology. *Oncotarget* **7**, 12748-12760, doi:10.18632/oncotarget.7220 (2016).

26 Balasubramanian, P. *et al*. Antibody-independent capture of circulating tumor cells of non-epithelial origin with the ApoStream(R) system. *PloS one* **12**, e0175414, doi:10.1371/journal.pone.0175414 (2017).

27 Gascoyne, P. R., Noshari, J., Anderson, T. J. & Becker, F. F. Isolation of rare cells from cell mixtures by dielectrophoresis. *Electrophoresis* **30**, 1388-1398, doi:10.1002/elps.200800373 (2009).

28 Shim, S. *et al*. Antibody-independent isolation of circulating tumor cells by continuous-flow dielectrophoresis. *Biomicrofluidics* **7**, 11807, doi:10.1063/1.4774304 (2013).

29 Antfolk, M., Antfolk, C., Lilja, H., Laurell, T. & Augustsson, P. A single inlet two-stage acoustophoresis chip enabling tumor cell enrichment from white blood cells. *Lab Chip* **15**, 2102-2109, doi:10.1039/c5lc00078e (2015).

30 Urbansky, A. *et al*. Rapid and effective enrichment of mononuclear cells from blood using acoustophoresis. *Scientific reports* **7**, 17161 (2017).

31 Augustsson, P., Magnusson, C., Nordin, M., Lilja, H. & Laurell, T. Microfluidic, label-free enrichment of prostate cancer cells in blood based on acoustophoresis. *Analytical chemistry* **84**, 7954-7962, doi:10.1021/ac301723s (2012).

32 Li, P. *et al*. Acoustic separation of circulating tumor cells. *Proceedings of the National Academy of Sciences of the United States of America* **112**, 4970-4975, doi:10.1073/pnas.1504484112 (2015).

33 Antfolk, M., Kim, S. H., Koizumi, S., Fujii, T. & Laurell, T. Label-free single-cell separation and imaging of cancer cells using an integrated microfluidic system. *Scientific reports* **7**, 46507, doi:10.1038/srep46507 (2017).

34 Lohr, J. G. *et al*. Genetic interrogation of circulating multiple myeloma cells at single-cell resolution. *Science translational medicine* **8**, 363ra147 (2016).

35 Rodrigues, M. A. Automation of the in vitro micronucleus assay using the Imagestream® imaging flow cytometer. *Cytometry Part A* **93**, 706-726 (2018).

36 Hillig, T., Nygaard, A.-B., Nekiunaite, L., Klingelhöfer, J. & Sölétormos, G. In vitro validation of an ultra-sensitive scanning fluorescence microscope for analysis of Circulating Tumor Cells. *APMIS* **122**, 545-551, doi:10.1111/apm.12183 (2014).

37 Payne, R. E. *et al.* Viable circulating tumour cell detection using multiplex RNA in situ hybridisation predicts progression-free survival in metastatic breast cancer patients. *British Journal Of Cancer* **106**, 1790, doi:10.1038/bjc.2012.137 (2012).

38 Werner, S. L. *et al.* Analytical Validation and Capabilities of the Epic CTC Platform: Enrichment-Free Circulating Tumour Cell Detection and Characterization. *Journal of circulating biomarkers* **4**, 3, doi:10.5772/60725 (2015).

39 Galanzha, E. I. & Zharov, V. P. Circulating Tumor Cell Detection and Capture by Photoacoustic Flow Cytometry in Vivo and ex Vivo. *Cancers* **5**, 1691-1738, doi:10.3390/cancers5041691 (2013).

40 Alix-Panabières, C. *et al.* Characterization and enumeration of cells secreting tumor markers in the peripheral blood of breast cancer patients. *Journal of Immunological Methods* **299**, 177-188, doi:https://doi.org/10.1016/j.jim.2005.02.007 (2005).

41 Paris, P. L. *et al.* Functional phenotyping and genotyping of circulating tumor cells from patients with castration resistant prostate cancer. *Cancer Letters* **277**, 164-173, doi:https://doi.org/10.1016/j.canlet.2008.12.007 (2009).

42 Cann, G. M. *et al.* mRNA-Seq of Single Prostate Cancer Circulating Tumor Cells Reveals Recapitulation of Gene Expression and Pathways Found in Prostate Cancer. *PloS one* **7**, e49144, doi:10.1371/journal.pone.0049144 (2012).

43 Park, S. M. *et al.* Molecular profiling of single circulating tumor cells from lung cancer patients. *Proceedings of the National Academy of Sciences of the United States of America* **113**, E8379-E8386, doi:10.1073/pnas.1608461113 (2016).

44 Powell, A. A. *et al.* Single cell profiling of circulating tumor cells: transcriptional heterogeneity and diversity from breast cancer cell lines. *PloS one* **7**, e33788, doi:10.1371/journal.pone.0033788 (2012).

45 Ramskold, D. *et al.* Full-length mRNA-Seq from single-cell levels of RNA and individual circulating tumor cells. *Nature biotechnology* **30**, 777-782, doi:10.1038/nbt.2282 (2012).

46 Po, J. W. *et al.* Improved ovarian cancer EMT-CTC isolation by immunomagnetic targeting of epithelial EpCAM and mesenchymal N-cadherin. *Journal of circulating biomarkers* **7**, 1849454418782617, doi:10.1177/1849454418782617 (2018).

47 Allard, W. J. *et al.* Tumor cells circulate in the peripheral blood of all major carcinomas but not in healthy subjects or patients with nonmalignant diseases. *Clin Cancer Res* **10**, 6897-6904, doi:10.1158/1078-0432.CCR-04-0378 (2004).

48 Zieglschmid, V. *et al.* Combination of Immunomagnetic Enrichment with Multiplex RT-PCR Analysis for the Detection of Disseminated Tumor Cells. *Anticancer research* **25**, 1803-1810 (2005).

49 Pluim, D., Devriese, L. A., Beijnen, J. H. & Schellens, J. H. M. Validation of a multiparameter flow cytometry method for the determination of phosphorylated extracellular-signal-regulated kinase and DNA in circulating tumor cells. *Cytometry Part A* **81A**, 664-671, doi:10.1002/cyto.a.22049 (2012).

50 Talasaz, A. H. *et al.* Isolating highly enriched populations of circulating epithelial cells and other rare cells from blood using a magnetic sweeper device. *Proceedings of the National Academy of Sciences of the United States of America* **106**, 3970-3975, doi:10.1073/pnas.0813188106 (2009).

51 Thege, F. I. *et al.* Microfluidic immunocapture of circulating pancreatic cells using parallel EpCAM and MUC1 capture: characterization, optimization and downstream analysis. *Lab on a chip* **14**, 1775-1784, doi:10.1039/c4lc00041b (2014).

52 Nora Dickson, M. *et al.* Efficient capture of circulating tumor cells with a novel immunocytochemical microfluidic device. *Biomicrofluidics* **5**, 34119-3411915, doi:10.1063/1.3623748 (2011).

53 Stott, S. L. *et al.* Isolation of circulating tumor cells using a microvortex-generating herringbone-chip. *Proceedings of the National Academy of Sciences* **107**, 18392, doi:10.1073/pnas.1012539107 (2010).

54 Harb, W. *et al.* Mutational Analysis of Circulating Tumor Cells Using a Novel Microfluidic Collection Device and qPCR Assay. *Translational Oncology* **6**, 528-IN521, doi:https://doi.org/10.1593/tlo.13367 (2013).

55 Nagrath, S. *et al.* Isolation of rare circulating tumour cells in cancer patients by microchip technology. *Nature* **450**, 1235-1239, doi:10.1038/nature06385 (2007).

56 Wang, S. *et al.* Highly Efficient Capture of Circulating Tumor Cells by Using Nanostructured Silicon Substrates with Integrated Chaotic Micromixers. *Angewandte Chemie International Edition* **50**, 3084-3088, doi:10.1002/anie.201005853 (2011).

57 Sheng, W. *et al*. Capture, release and culture of circulating tumor cells from pancreatic cancer patients using an enhanced mixing chip. *Lab on a chip* **14**, 89-98, doi:10.1039/c3lc51017d (2014).

58 Yoon, H. J. *et al*. Sensitive capture of circulating tumour cells by functionalized graphene oxide nanosheets. *Nature nanotechnology* **8**, 735-741, doi:10.1038/nnano.2013.194 (2013).

59 Kamande, J. W. *et al*. Modular microsystem for the isolation, enumeration, and phenotyping of circulating tumor cells in patients with pancreatic cancer. *Analytical chemistry* **85**, 9092-9100, doi:10.1021/ac401720k (2013).

60 Autebert, J. *et al*. High purity microfluidic sorting and analysis of circulating tumor cells: towards routine mutation detection. *Lab on a chip* **15**, 2090-2101, doi:10.1039/C5LC00104H (2015).

61 Earhart, C. M. *et al*. Isolation and mutational analysis of circulating tumor cells from lung cancer patients with magnetic sifters and biochips. *Lab on a chip* **14**, 78-88, doi:10.1039/c3lc50580d (2014).

62 Winer-Jones, J. P. *et al*. Circulating tumor cells: clinically relevant molecular access based on a novel CTC flow cell. *PloS one* **9**, e86717, doi:10.1371/journal.pone.0086717 (2014).

63 Liu, Z. *et al*. Negative enrichment by immunomagnetic nanobeads for unbiased characterization of circulating tumor cells from peripheral blood of cancer patients. *Journal of translational medicine* **9**, 70, doi:10.1186/1479-5876-9-70 (2011).

64 Meye, A. *et al*. Isolation and enrichment of urologic tumor cells in blood samples by a semi-automated CD45 depletion autoMACS protocol. *International journal of oncology* **21**, 521-530 (2002).

65 Naume, B. *et al*. Immunomagnetic techniques for the enrichment and detection of isolated breast carcinoma cells in bone marrow and peripheral blood. *Journal of hematotherapy* **6**, 103-114, doi:10.1089/scd.1.1997.6.103 (1997).

66 Wu, S. *et al*. Enrichment and enumeration of circulating tumor cells by efficient depletion of leukocyte fractions. *Clinical chemistry and laboratory medicine* **52**, 243-251, doi:10.1515/cclm-2013-0558 (2014).
